# Central nervous system biomarkers GFAp and NfL associate with post-acute cognitive impairment and fatigue following critical COVID-19

**DOI:** 10.1038/s41598-023-39698-y

**Published:** 2023-08-12

**Authors:** Lovisa Bark, Ing-Marie Larsson, Ewa Wallin, Joel Simrén, Henrik Zetterberg, Miklos Lipcsey, Robert Frithiof, Elham Rostami, Michael Hultström

**Affiliations:** 1 Anaesthesiology and Intensive Care Medicine, Department of Surgical Sciences, Uppsala University Hospital, Uppsala University, Entr. 70, Floor 2, 75185 Uppsala, Sweden; 2 Department of Psychiatry and Neurochemistry, Institute of Neuroscience and Physiology, The Sahlgrenska Academy at the University of Gothenburg, Mölndal, Sweden; 3 Clinical Neurochemistry Laboratory, Sahlgrenska University Hospital, Mölndal, Sweden; 4 Department of Neurodegenerative Disease, UCL Institute of Neurology, Queen Square, London, UK; 5 UK Dementia Research Institute at UCL, London, UK; 6 Hong Kong Center for Neurodegenerative Diseases, Clear Water Bay, Hong Kong China; 7 Hedenstierna Laboratory, Department of Surgical Sciences, Uppsala University, Uppsala, Sweden; 8 Department of Neuroscience, Karolinska Institutet, Stockholm, Sweden; 9 Department of Medical Sciences, Uppsala University, Uppsala, Sweden; 10 Integrative Physiology, Department of Medical Cell Biology, Uppsala University, Uppsala, Sweden; 11 Department of Epidemiology, Biostatistics and Occupational Health, McGill University, Montréal, QC Canada; 12 Lady Davis Institute of Medical Research, Jewish General Hospital, Montréal, QC Canada

**Keywords:** Viral infection, Biomarkers

## Abstract

A high proportion of patients with coronavirus disease 2019 (COVID-19) experience post-acute COVID-19, including neuropsychiatric symptoms. Objective signs of central nervous system (CNS) damage can be investigated using CNS biomarkers such as glial fibrillary acidic protein (GFAp), neurofilament light chain (NfL) and total tau (t-tau). We have examined whether CNS biomarkers can predict fatigue and cognitive impairment 3–6 months after discharge from the intensive care unit (ICU) in critically ill COVID-19 patients. Fifty-seven COVID-19 patients admitted to the ICU were included with analysis of CNS biomarkers in blood at the ICU and at follow up. Cognitive dysfunction and fatigue were assessed with the Montreal Cognitive Assessment (MoCA) and the Multidimensional Fatigue inventory (MFI-20). Elevated GFAp at follow-up 3–6 months after ICU discharge was associated to the development of mild cognitive dysfunction (*p* = 0.01), especially in women (*p* = 0.005). Patients who experienced different dimensions of fatigue at follow-up had significantly lower GFAp in both the ICU and at follow-up, specifically in general fatigue (*p* = 0.009), physical fatigue (*p* = 0.004), mental fatigue (*p* = 0.001), and reduced motivation (*p* = 0.001). Women showed a more pronounced decrease in GFAp compared to men, except for in mental fatigue where men showed a more pronounced GFAp decrease compared to women. NfL concentration at follow-up was lower in patients who experienced reduced motivation (*p* = 0.004). Our findings suggest that GFAp and NfL are associated with neuropsychiatric outcome after critical COVID-19.

*Trial registration* The study was registered à priori (clinicaltrials.gov: NCT04316884 registered on 2020-03-13 and NCT04474249 registered on 2020-06-29).

## Introduction

The coronavirus disease 2019 (COVID-19) pandemic is primarily known to cause respiratory failure and severe disease necessitating intensive care. It is common that severely ill patients develop symptoms from both the central nervous system (CNS) and the peripheral nervous system (PNS)^1^. This is particularly true for critically ill COVID-19 patients treated in an intensive care unit (ICU)^2^. At follow-up 2–3 months from onset of COVID-19 a high proportion of patients experience long-term symptoms, called post-acute COVID-19^3–5^. These include fatigue, affecting 31–69% of patients^6–8^, and cognitive impairment, affecting 21–50% of patients^9,10^. In critically ill non-COVID-19 patients with acute respiratory distress syndrome (ARDS) the development of the post-intensive care syndrome (PICS) it is also common with new or worsening physical, cognitive and mental impairments^11^, affecting 56–64% of patients^12^.

Thus, COVID-19 is associated with persistent CNS symptoms that could be due to cellular damage. However, the exact mechanisms remain unknown. With CNS biomarkers it is possible to objectively assess nerve cell and glial injury. In our study we used the CNS biomarkers glial fibrillary acidic protein (GFAp) as a biomarker for astrocytic damage^13^, neurofilament light chain (NfL) as a biomarker for axonal damage^14^, and total tau (t-tau), as a biomarker for neuronal damage^15^. These CNS biomarkers have been found to be elevated in hospitalized COVID-19 patients and associated with the development of critical illness polyneuropathy (CIN) and critical illness myopathy (CIM)^2^. Raised values of NfL in hospitalized COVID-19 patients have been related to the need for mechanical ventilation, prolonged ICU stay/hospitalisation and worse outcome^16^. Also, NfL values were higher in COVID-19 patients compared to non-COVID-19 patients in the ICU after adjustment for age and pre-existing comorbidities. Regardless of COVID/non-COVID status, elevated NfL was associated with unfavourable outcome^17^. Initially elevated NfL and GFAp normalized after 6 months, suggesting that the neurological symptoms were not due to ongoing CNS injury^18,19^. However, the relationship between CNS biomarkers and cognitive impairment and fatigue has not yet been widely studied. Our hypothesis was that patients with critical COVID-19 have elevated biomarkers that can be associated to the development of neuropsychiatric symptoms at follow-up.

The aim of this study was to investigate whether the CNS biomarkers NfL, GFAp and t-tau are associated to fatigue measured with the Multidimensional Fatigue Inventory-20 (MFI-20), as well as cognitive impairment measured with the Montreal Cognitive Assessment (MoCA), at 3–6 months after ICU discharge in critically ill COVID-19 patients.

## Methods

### Study design

This is a sub-study of an ongoing prospective observational study being performed in the general ICU at the Uppsala University Hospital in Sweden, approved by the National Ethical Review Agency (Dnr 2017/043 (with amendments 2019-00169, 2020-01623, 2020-02719, 2020-05730, 2021-01469), as well as the de-novo application Dnr 2022-00526-01 pertaining to the acute study, and Dnr 2020-02697 (with amendments 2020-03629, 2020-05758, 2021-02205, 2022-01115-02) pertaining to the follow-up study). The study was listed at ClinicalTrials.gov (NCT03720860). During the intensive care period informed consent was obtained from the patient or next of kin if the patient was unable to give consent, and all patients gave written informed consent to participate in the follow-up study. The Declaration of Helsinki and its subsequent revisions were followed.

### Subjects

**Figure 1:**
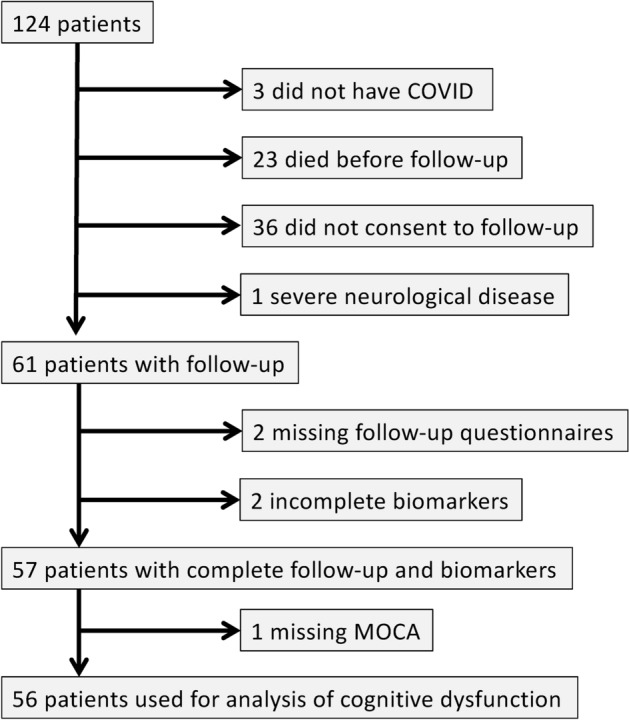
Flowchart over enrolled patients. A total of 121 critical ill COVID-19 patients were included when admitted to the ICU. Three patients were later PCR-confirmed COVID-19 negative. Twenty-three patients died before follow-up and 36 patients did not consent to follow-up. One patient was excluded due to severe neurological disease. Sixty-one patients were available at follow-up 3–6 months after discharge from the ICU, 2 patients had incomplete follow-up questionnaires and 2 patients had incomplete biomarker analysis. A total of 57 patients had complete follow-up and biomarker data, however one patient had incomplete MoCA therefore 56 patients were available for cognitive impairment analysis. *ICU* Intensive care unit. *MoCA* Montreal cognitive assessment score.

All adult patients with confirmed COVID-19 infection by nasopharyngeal polymerase chain reaction (PCR) admitted to the ICU between 14th of March to 8th of September 2020 were screened for inclusion. Exclusion criteria were limited to patients without COVID-19 and patients who were unable to attend, or did not consent, to follow up. In accordance with the guidelines of the Swedish society of anaesthesiology and intensive care (SFAI), patients with severe pre-existing cognitive impairment were not eligible for admission to the intensive care unit and thus none of the included patients had known pre-existing cognitive impairment. However, one patient with a severe neurological disease had known risk for pre-existing fatigue and was therefore excluded (Fig. 1).

Risk for in hospital mortality was evaluated at admission to the ICU using Simplified acute physiology score 3 (SAPS3) which ranges from 16 to 217 points with higher points indicating higher risk of mortality^20^. EDTA plasma was collected at the ICU within the first days of admission and again at 3–6 months after discharge from the ICU, and frozen at − 80 °C until use.

At follow-up, 3–6 months after the ICU stay, patients were assessed regarding fatigue using MFI-20^21^ and cognitive function using MoCA^22^. The MFI-20 questionnaire consists of a total of 20 items in five subscales with each subscale containing its own aspect of fatigue; general fatigue, physical fatigue, mental fatigue, reduced activity and reduced motivation. High scores represent a high level of fatigue and each subscale range from 4–20 points^21^. For MFI-20, a score corresponding to the ≥ 95 percentile in a normal population stratified by age and gender was used to identify patients with significant fatigue^21^, and the patient cohort was subsequently classified as having fatigue or not having fatigue for those without fatigue at follow-up.

The MoCA questionnaire has a high sensitivity and specificity to detect mild cognitive dysfunction^22^. It assesses cognitive function through 10 items over several cognitive domains. For cognitive assessment a cut-off value lower than 26 points on MoCA (max 30 points) was used to indicate cognitive impairment and 26 points or above to indicate no cognitive impairment. Education level affects the total score and therefore an additional point is added to correct for education of less than 12 years^22^.

In the figures the patients are presented by post-COVID status into “no fatigue”, and “fatigue”, and reported separately for “ICU” and “follow-up” resulting in a total of four groups. We also examined the relationship between gender and the development of fatigue by presenting the patients grouped as “no fatigue female” or “fatigued female” and “no fatigue male” or “fatigued male”. Similarly, the statistical analysis of cognitive impairment was based on a linear mixed model of biomarker concentration by cognitive classification taking gender and age into consideration, and the figures are presented separately showing the association for cognitive status by analysis time-point, and by gender.

### CNS biomarker analysis

Analysis of NfL, t-tau and GFAp was performed at the Clinical Neurochemistry Laboratory at Sahlgrenska University Hospital using single molecule array (Simoa) technology (Quanterix, Billerica, MA) and a single batch of reagents^18^. Intra-assay coefficients for internal control samples were below 7% for all analytes. The lower limit of quantification for GFAp was 30.9 pg/ml and in this study 15 samples were below that threshold. For NfL, the limit was 2.21 pg/ml with no samples being below that threshold. Finally, for t-tau, the threshold was 0.39 pg/ml and three samples were below that threshold.

### Statistics

Continuous variables are presented as mean (standard deviation), frequencies/categorical variables are presented as an absolute number (percent of total study population), biomarker concentration variables are presented as median (interquartile range). The patient characteristics of the analyzed sample was compared with the whole cohort using T-tests for continuous variables, and Chi-square or Fisher’s exact test for categorical variables. We used a linear mixed model of biomarker concentration by fatigue classification taking gender and age into consideration. The statistical analysis of biomarker association with fatigue was based on a repeated measures mixed model multivariable ANOVA taking age and gender into account, testing whether each of the biomarkers were associated with any of the outcomes. Biomarker concentrations were log transformed to normalize their distribution. A *p*-value < 0.05 was considered significant. The statistical analysis was made using R (version 4.03).

### Ethics approval and consent to participate

The National Ethical Review Agency approved the study Dnr 2017-043 (with amendments 2019-00169, 2020-01623, 2020-02719, 2020-05730, 2021-01469), as well as de-novo application Dnr 2022-00526-01 pertaining to the acute study, and Dnr 2020-02697 (with amendments 2020-03629, 2020-05758, 2021-02205, 2022-01115-02) pertaining to the follow-up study. The Declaration of Helsinki and its subsequent revisions were followed. Written informed consent was obtained from the patients when possible. Otherwise, informed consent was first asked from next to kin and later confirmed by patients if feasible. The study was registered à priori (clinicaltrials.gov: NCT04316884 registered on 2020-03-13 and NCT04474249 registered on 2020-06-29).

## Results

After exclusion of three PCR-negative patients, a total of 121 adult patients were included during the acute phase of the illness (Fig. 1). Of those, 23 patients died before follow-up and 36 patients did not consent to follow-up and they were therefore excluded. One patient was excluded because of pre-existing severe neurological disease. Additionally, 2 patients had incomplete follow-up questionnaires and 2 patients had incomplete biomarker analysis. This meant that 57 patients participated in MFI-20 and subsequent fatigue analysis. One additional missing MoCA questionnaire meant that 56 patients could be included in the cognitive dysfunction analysis.

The follow-up group is largely representative for the whole cohort at admission (Table 1). The majority was male, 77%, the average BMI was 30 ± 6 kg/m^2^ and age was 61 ± 14 years. Average day of admission after COVID-19 infection was 11 ± 4 days and average SAPS3 was 53 ± 10. At admission the majority of patients were fully awake, but a few were sedated at admission due to acute intubation in the emergency department. The most common comorbidities were hypertension, diabetes mellitus type 2, and pulmonary disease with no significant difference between admission and follow-up groups. A small number of patients had previous neurological disease, 6 patients (5%) in total and 1 patient (1.8%) in the follow-up group. The whole cohort had a total of 16 patients (13%) with psychiatric disease. In the follow up group 3 patients (5%) had psychiatric disease (Table 1).

**Table 1:** Patient demographic characteristics and comorbidities in critically ill COVID-19 patients.

	All	Follow-up	*p*-value
N	121	57	
Women	28 (23%)	14 (25%)	NS
Age (years)	61 (14)	60 (13)	NS
BMI (kg/m^2^)	30 (6)	30 (6)	NS
COVID-19 day on ICU admission	11 (4)	11 (4)	NS
SAPS3	53 (10)	51 (9)	NS
Temperature at admission©)	38 (1)	38 (1)	NS
Breathing rate at admission	30 (9)	30 (9)	NS
Vasoactive treatment	7 (6%)	2 (4%)	NS
Sedated at admission	4 (3%)	0 (0%)	NS
Hypertension	64 (53%)	26 (46%)	NS
Heart failure	5 (4%)	0 (0%)	NS
Ischemic heart disease	13 (11%)	2 (4%)	NS
Diabetes mellitus	33 (27%)	10 (18%)	NS
Neurological disease	6 (5%)	1 (1.8%)	NS
Psychiatric disease	16 (13%)	3 (5%)	NS
Pulmonary disease	33 (27%)	15 (26%)	NS

Overview of all included patients and the follow-up group. In the follow-up group there are generally fewer comorbidities except for pulmonary disease. Otherwise, the follow-up group was largely representative for the whole cohort at admission. Variables are presented as average (standard deviation) or as number (percentage of total). *BMI* Body mass index, *ICU* Intensive care unit. *SAPS3* Simplified acute physiology score 3. *NS* no significance.

Similarly, there was no significant difference between the follow-up group and the cohort at discharge regarding complications during the ICU stay such as thrombotic events, delirium and critical illness polyneuropathy/critical illness myopathy (Table 2). Fourteen patients (12%) in total and 6 (11%) of the follow-up patients had a thrombotic event, and 14 patients (12%) of the total and 6 (11%) of the follow-up patients developed CIN/CIM. The median length of ICU stay for the whole cohort was 8 (5–16) days. Fifty-five percent of the patients were subjected to invasive ventilation and vasoactive treatment, lasting 2 (0–9) days and 2 (0–7) days respectively in the whole cohort compared to the follow-up group where invasive ventilation lasted 0 (0–6) days and vasoactive treatment 1 (0–8) days.

**Table 2:** Discharge parameters for critically ill COVID-19 patients. Overview of all included patients and the 3–6 months follow-up group.

	All	Follow-up	*p*-value
N	121	57	
Length of ICU stay in days	8 (5–16)	9 (5–15)	NS
Alive at follow-up	98 (81%)	57 (100%)	
Thrombotic event	14 (12%)	6 (11%)	NS
1. Myocardial infarction	2 (1.7%)	1 (1.8%)	NS
2. Pulmonary embolism	10 (8%)	4 (7%)	NS
3. Stroke	4 (3%)	2 (4%)	NS
4. Deep vein thrombosis	1 (0.8%)	1 (1.8%)	NS
Critical illness polyneuropathy/critical illness myopathy	14 (12%)	6 (11%)	NS
Delirium	10 (8%)	6 (11%)	NS
Days with vasoactive treatment	2 (0–7)	0 (0–6)	NS
Number of patients with vasoactive treatment	68 (56%)	28 (49%)	NS
Days with ventilation	2 (0–9)	1 (0–8)	NS
Number of patients with mechanical ventilation	67 (55%)	31 (54%)	NS

The follow-up group was largely representative for the whole cohort at discharge regarding complications during the ICU stay such as thrombotic events, delirium and critical illness polyneuropathy/critical illness myopathy. Roughly equally many patients were subjected to invasive ventilation and vasoactive treatment. Variables are presented as average (standard deviation), number (percentage of total) or as median (interquartile range). *ICU* Intensive care unit. *NS* no significance.

### Fatigue and cognitive function

A total of 57 patients filled out the MFI-questionnaire and a total of 56 patients filled out the MoCA-questionnaire. Analysis of admission (Table 3) and discharge (Table 4) parameters showed no significant difference between the groups with and without fatigue or between the groups with and without cognitive impairment. A total of 24 patients had mild cognitive impairment with scores within the range of 18–25 p. One patient had moderate cognitive impairment with a score within the range of 10–17 p. No patients had severe cognitive impairment. Twenty-three patients received 1 p for having 12 or less years of education (Table 5).

**Table 3:** Patient demographic characteristics and comorbidities in critically ill COVID-19 patients.

	Fatigue	No fatigue	*p*-value	Cognitive impairment	No cognitive impairment	*p*-value
N	31	26		25	32	
Women	6 (19%)	8 (31%)	NS	7 (28%)	7 (22%)	NS
Age (years)	57 (15)	63 (10)	NS	60 (12)	59 (13)	NS
BMI (kg/m^2^)	31 (7)	28 (5)	NS	31 (6)	29 (5)	NS
COVID-19 day on ICU admission	11 (5)	10 (3)	NS	11 (4)	11 (4)	NS
SAPS3	50 (9)	52 (8)	NS	52 (9)	51 (9)	NS
Temperature at admission©)	38 (1)	38 (1)	NS	38 (1)	38 (1)	NS
Breathing rate at admission	31 (9)	28 (8)	NS	28 (8)	30 (9)	NS
Vasoactive treatment	1 (3%)	1 (4%)	NS	1 (4%)	1 (3%)	NS
Sedated at admission	0 (0%)	0 (0%)	NS	0 (0%)	0 (0%)	NS
Hypertension	15 (48%)	11 (42%)	NS	11 (44%)	15 (47%)	NS
Heart failure	0 (0%)	0 (0%)	NS	0 (0%)	0 (0%)	NS
Ischemic heart disease	1 (3%)	1 (4%)	NS	1 (4%)	1 (3%)	NS
Diabetes mellitus	5 (16%)	5 (19%)	NS	5 (20%)	5 (16%)	NS
Neurological disease	1 (3%)	0 (0%)	NS	0 (0%)	1 (3%)	NS
Psychiatric disease	2 (6%)	1 (4%)	NS	3 (12%)	0 (0%)	NS
Pulmonary disease	10 (32%)	5 (19%)	NS	6 (24%)	9 (29%)	NS

Overview of admission parameters of patients with fatigue, without fatigue, with cognitive impairment, and without cognitive impairment at follow up. No significant differences were found in admission parameters. Variables are presented as average (standard deviation) or as number (percentage of total). *BMI* Body mass index. *ICU* Intensive care unit. *SAPS3* Simplified acute physiology score 3. *NS* no significance.

**Table 4:** Discharge parameters for the critically ill COVID-19 patients divided into groups with fatigue, without fatigue, with cognitive impairment and without cognitive impairment.

	Fatigue	No fatigue	*p*-value	Cognitive impairment	No cognitive impairment	*p*-value
N	31	26		25	32	
Length of ICU stay in days	9 (5–14.5)	9.5 (6–15.25)	NS	9 (5–12)	10 (5–16)	NS
Thrombotic event	3 (10%)	3 (12%)	NS	3 (12%)	3 (9%)	NS
1. Myocardial infarction	1 (4%)	1 (4%)	NS	1 (4%)	1 (3%)	NS
2. Pulmonary embolism	1 (4%)	0 (0%)	NS	0 (0%)	1 (3%)	NS
3. Stroke	2 (8%)	2 (8%)	NS	1 (4%)	3 (9%)	NS
4. Deep vein thrombosis	1 (4%)	0 (0%)	NS	1 (4%)	0 (0%)	NS
Critical illness polyneuropathy/	4 (13%)	2 (8%)	NS	2 (8%)	3 (9%)	NS
critical illness myopathy						
Delirium	3 (10%)	3 (12%)	NS	2 (8%)	4 (13%)	NS
Days with vasoactive treatment	0 (0–6.5)	1 (0–4.75)	NS	2 (0–5)	0 (0–7)	NS
Number of patients with vasoactive treatment	14 (45%)	13 (50%)	NS	11 (44%)	16 (49%)	NS
Days with ventilation	1 (0–8.5)	1.5 (0–6)	NS	3 (0–6)	0.5 (0–8.25)	NS
Number of patients with mechanical ventilation	14 (45%)	13 (50%)	NS	11 (44%)	16 (50%)	NS

No significant differences were found in any of the discharge parameters. Variables are presented as average (standard deviation), number (percentage of total) or as median (interquartile range). *ICU* Intensive care unit. *NS* no significance.

**Table 5:** Results of the MFI-20 subscales and MoCA 3–6 months after discharge from the ICU in 57 critically ill COVID-19 patients.

	Mean score	Worse than cutoff
MFI general fatigue	14 (5)	26 (46%)
MFI physical fatigue	13 (5)	20 (35%)
MFI mental fatigue	11 (5)	15 (26%)
MFI reduced motivation	10 (4)	10 (18%)
MFI reduced activity	13 (5)	19 (33%)
MoCA	26 (3)	25 (45%)

MFI-20 results are divided in the subscales general fatigue, physical fatigue, mental fatigue, reduced motivational and reduced activity. The cutoff value means worse than a normal population 95th percentile calculation based on gender and age. MoCA score lower than 26 indicates at least mild cognitive dysfunction. One patient had an incomplete MoCA-questionnaire therefor are the values based on 56 patients for the MoCA. Scores presented as mean (standard deviation) and cutoff presented as an absolute number (percent of total). *MFI* Multidimensional Fatigue inventory. *MOCA* Montreal Cognitive Assessment. *ICU* Intensive care unit.

### CNS biomarker analysis

**Figure 2:**
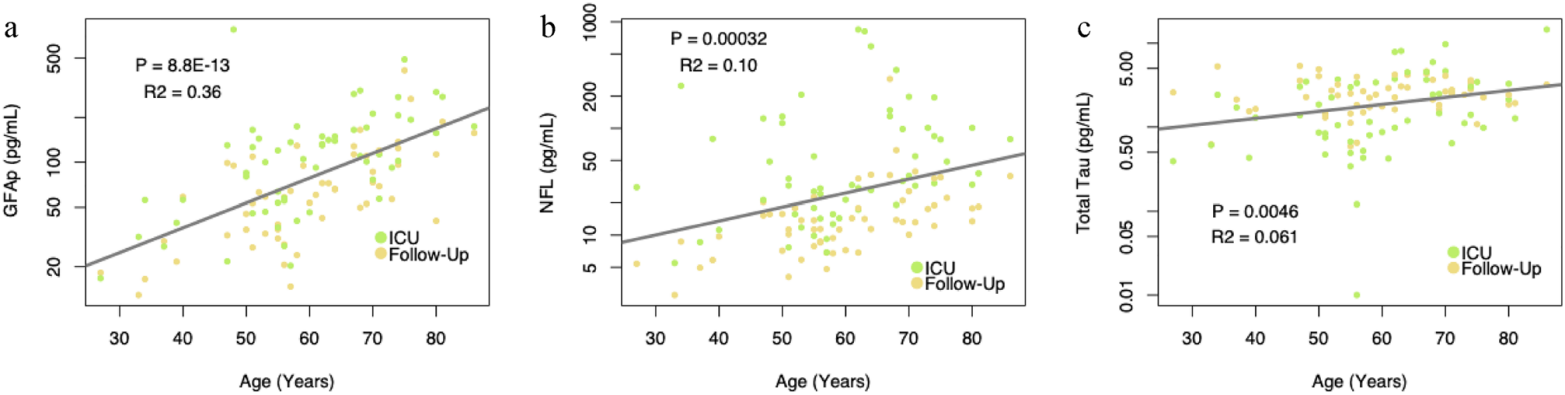
The CNS biomarkers GFAp, NfL and t-tau in correlation to age. (**a**) GFAp concentration correlation to age. GFAp levels increases with increasing age. (**b**) NfL concentration correlation to age. NfL levels increases with increasing age. (**c**) T-tau concentration correlation to age. T-tau levels increases slightly with increasing age. *ICU* intensive care unit. *GFAp* Glial fibrillary acidic protein. *NfL* Neurofilament light chain.

The dataset includes 57 patients with plasma samples of the three CNS biomarkers NfL, t-tau and GFAp, analyzed during acute COVID-19 at the ICU and at follow-up 3–6 months after discharge (Table 6). All biomarkers showed correlations with increased patient age, NfL (*p* < 0.001, R^2^ = 0.10), t-tau (*p-*value = 0.005, R^2^ = 0.06) but the effect was by far the strongest for GFAp (*p* < 0.001, R^2^ = 0.36) (Fig. 2). This was taken into account in the ANOVA as described above.

**Table 6:** CNS biomarker concentration for 57 critically ill COVID-19 patients in the ICU and at follow-up at 3–6 months after discharge. GFAp and NfL decreases over time whereas t-tau increases.

	N = 57	N = 57
CNS biomarker concentration	ICU	3–6 months after ICU
GFAp (pg/ml)	110 (56–165)	60 (36–110)
NfL (pg/ml)	31 (18–101)	14 (9–20)
T-tau (pg/ml)	1.5 (0.8–3.4)	2.4 (1.7–3.3)

Variables are presented as median (interquartile range). *CNS* Central nervous system. *ICU* intensive care unit. *GFAp* Glial fibrillary acidic protein. *NfL* Neurofilament light chain. *T*-*tau* total tau.

### Association between CNS biomarkers and fatigue

**Figure 3:**
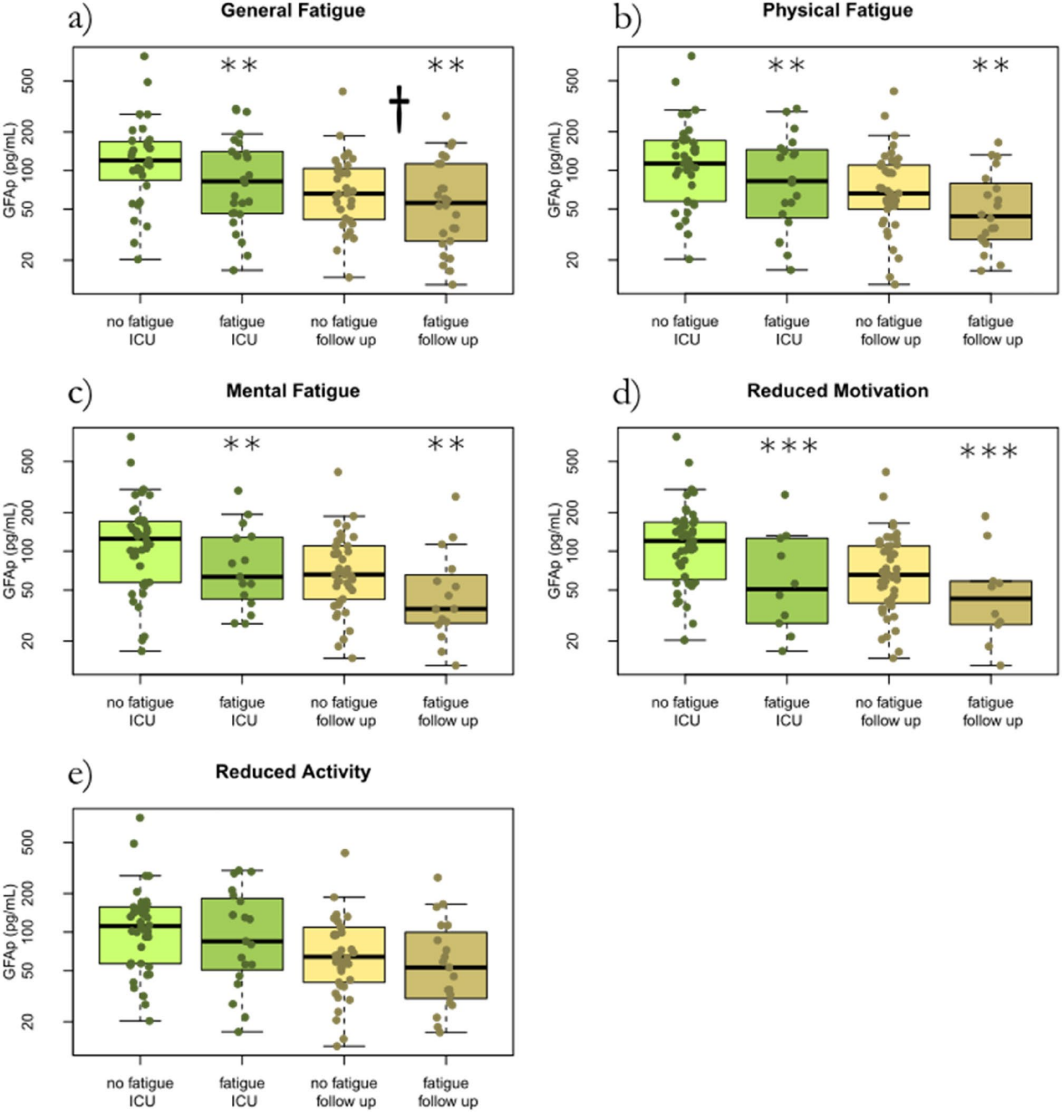
The glial biomarker GFAp concentration at ICU and at follow-up after 3–6 months in correlation to the MFI-20 dimensions of fatigue in 57 critically ill COVID-19 patients. No fatigue ICU, fatigue ICU, no fatigue follow-up, and fatigue follow-up, reflects fatigue status and whether GFAp was collected at the ICU or at follow-up. GFAp is higher during acute COVID-19 and decreases at follow-up (†). For general fatigue, physical fatigue, mental fatigue, and reduced motivation, GFAp is lower in those with fatigue. *GFAp* Glial fibrillary acidic protein. *ICU* intensive care unit. *MFI-20* Multidimensional Fatigue inventory.

**Figure 4:**
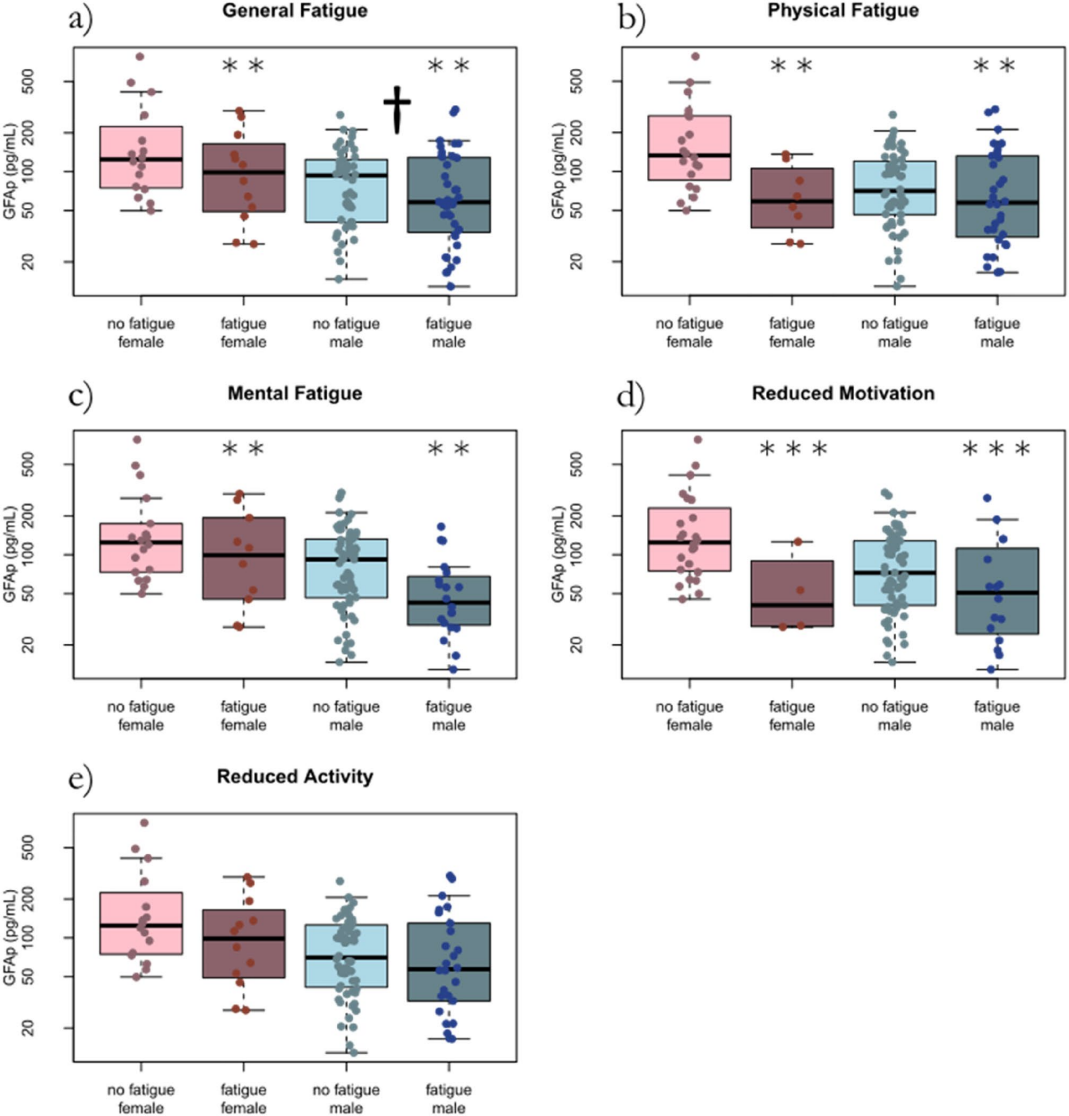
GFAp concentration in relation to gender. No fatigue female, fatigue female, no fatigue male, and fatigue male, reflects fatigue status and gender. GFAp is higher during acute COVID-19 and show significant effects in gender, being higher in women (†). For general fatigue, physical fatigue, mental fatigue, and reduced motivation, GFAp is lower in those with fatigue. Additionally, the effect is more pronounced in women, except for mental fatigue where it is more pronounced in men. *GFAp* Glial fibrillary acidic protein. *ICU* intensive care unit. *MFI-20* Multidimensional Fatigue inventory.

The average concentration of the glial biomarker GFAp was higher during acute COVID-19 compared to follow-up. GFAp showed significant effects for age and gender, being higher with increasing age and female gender. GFAp concentrations were lower both in the ICU and at follow-up in those with fatigue in the dimensions general fatigue (*p* = 0.009), physical fatigue (*p* = 0.004), mental fatigue (*p* = 0.001), and reduced motivation (*p* < 0.001) (Fig. 3 and Fig. 4). The effect was more pronounced in women, except for mental fatigue where it was more pronounced in men.

**Figure 5:**
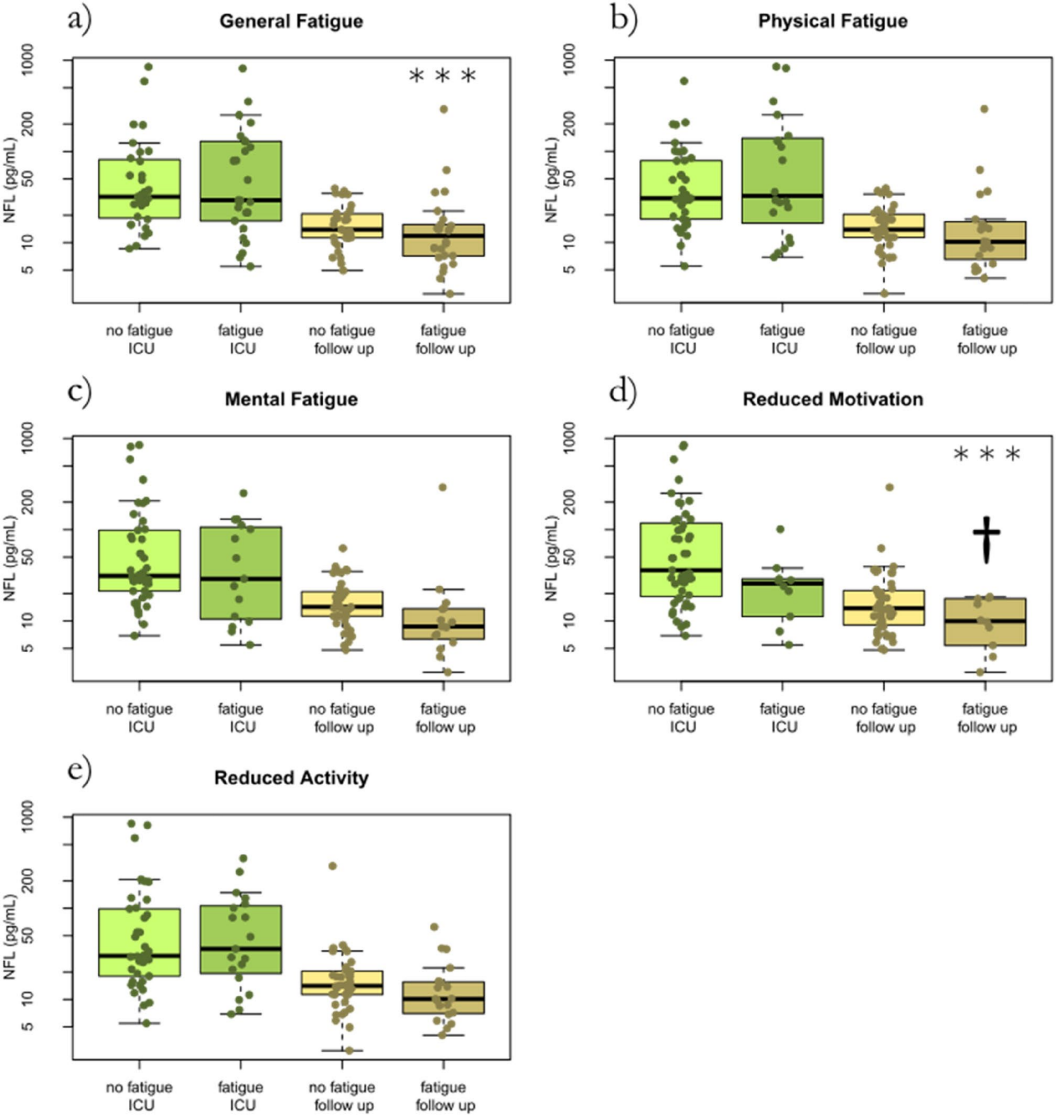
The axonal biomarker NfL concentration at ICU and at follow-up after 3–6 months in correlation to the MFI-20 dimensions of fatigue in 57 critical ill COVID-19 patients. No fatigue ICU, fatigue ICU, no fatigue follow-up, and fatigue follow-up, reflects fatigue status and whether NfL was collected at the ICU or at follow-up. NfL is high during acute critical COVID-19 and decreases to follow-up but show no strong relation to fatigue in the dimensions general fatigue, physical fatigue, mental fatigue or reduced activity. Patients experiencing reduced motivation (†) actually show a lower concentration. *ICU* intensive care unit. *NfL* Neurofilament light chain. *MFI-20* Multidimensional Fatigue inventory.

The average concentration of the axonal biomarker NfL was higher during the acute illness compared to follow-up (*p* < 0.001). There was no strong relationship to fatigue in the dimensions general fatigue, physical fatigue, mental fatigue, or reduced activity, other than for patients experiencing reduced motivation where the biomarker was lower at follow-up (*p* = 0.004) (Fig. 5).

**Figure 6:**
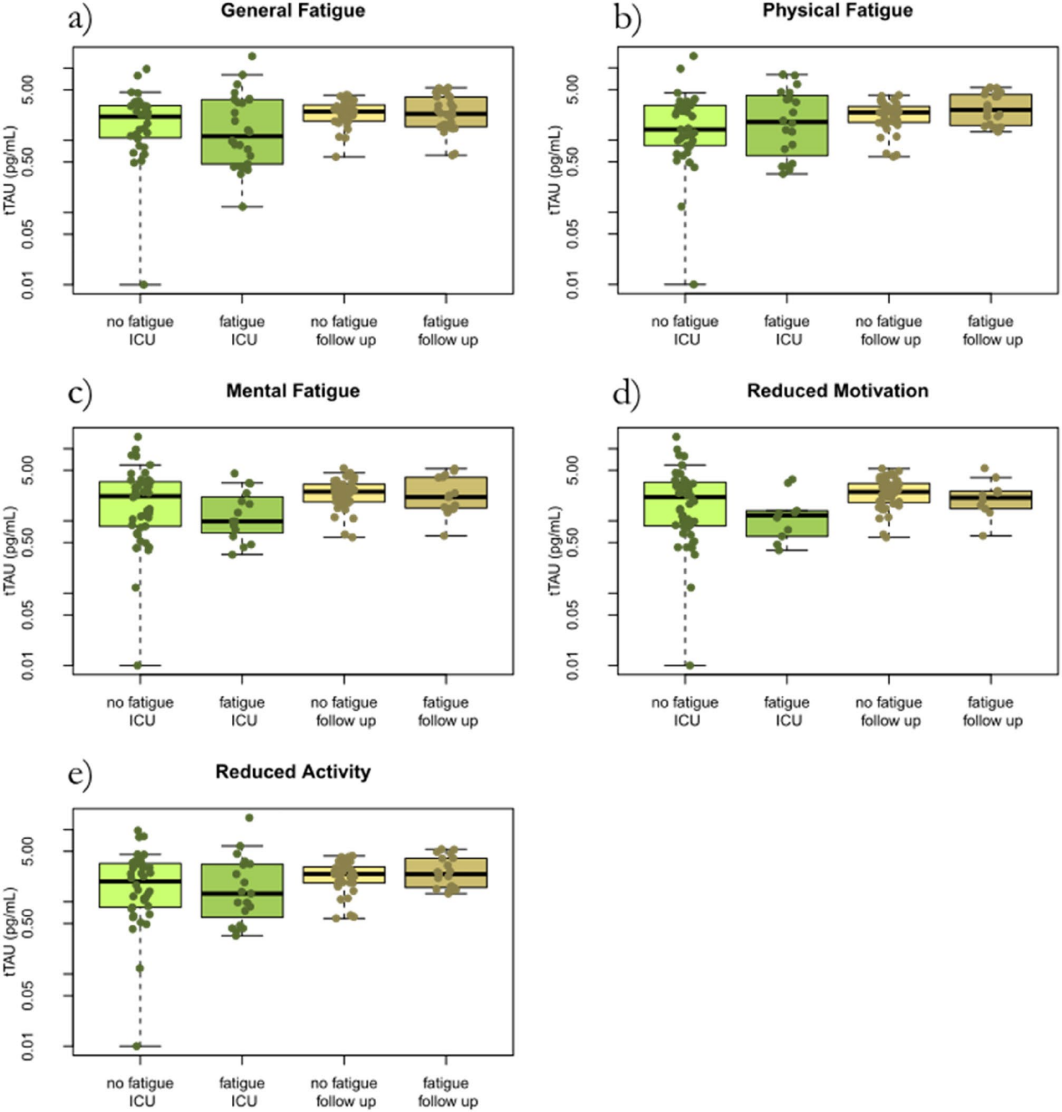
The neuronal biomarker t-tau concentration at ICU and at follow-up after 3–6 months in correlation to the MFI-20 dimensions of fatigue in 57 critical ill COVID-19 patients. No fatigue ICU, fatigue ICU, no fatigue follow-up, and fatigue follow-up, reflects fatigue status and whether the t-tau was collected at the ICU or at follow-up. T-tau concentration is slightly higher at follow-up compared to acute critical COVID-19 but large variation. No association to level of fatigue in any dimension. *T-tau* total-tau. *ICU* intensive care unit. *MFI-20* Multidimensional Fatigue inventory.

The average concentration of the neuronal biomarker t-tau was significantly higher at follow-up compared to acute critical COVID-19 but there were large variations in t-tau concentrations (*p* = 0.008). There was no association between t-tau and level of fatigue in any dimension, general, physical, mental, motivation or activity (Fig. 6).

### Association between CNS biomarkers and cognitive impairment

**Figure 7:**
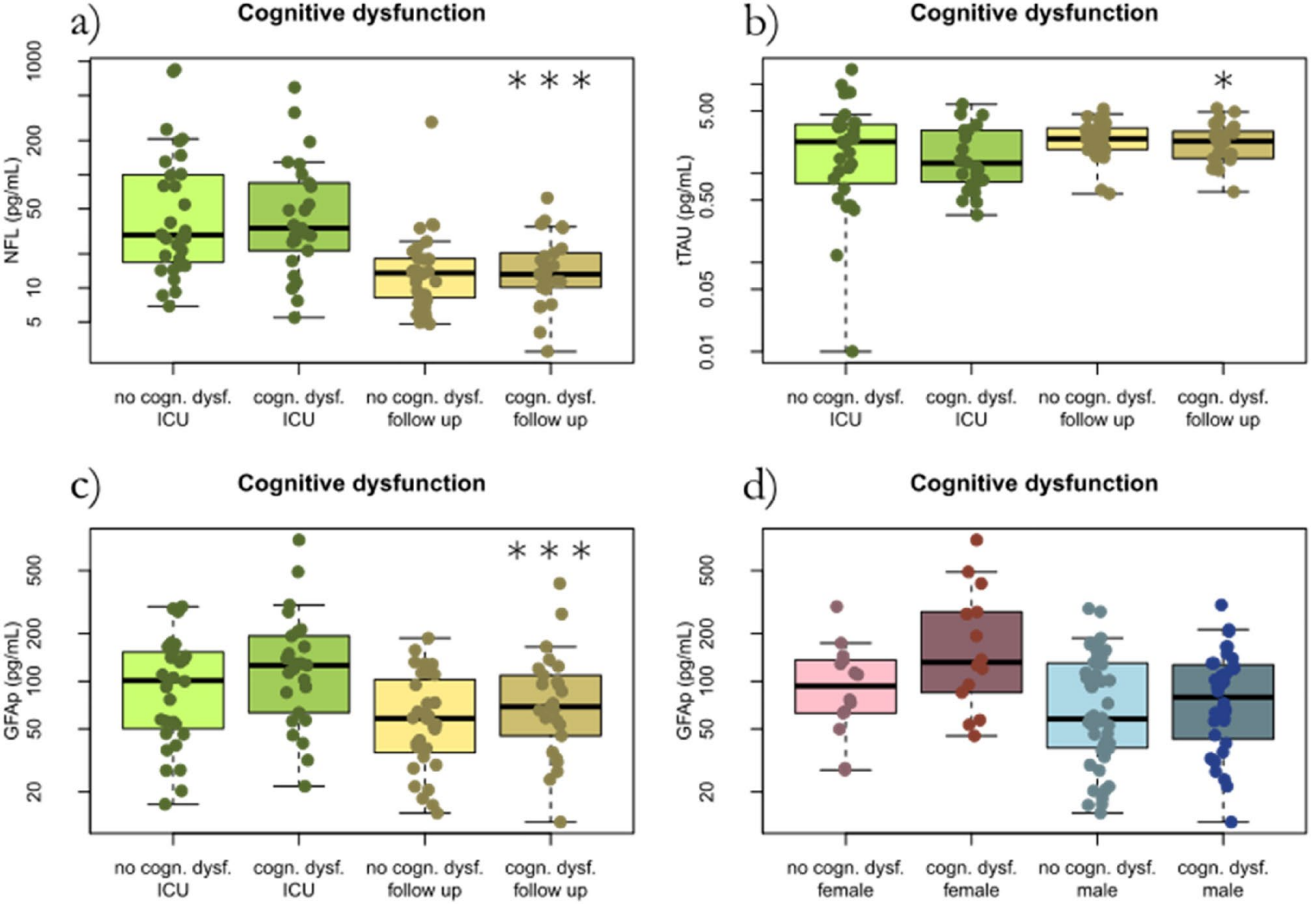
The biomarkers NfL, t-tau and GFAp concentrations at ICU and at follow-up after 3–6 months in correlation to MoCA assessing cognitive dysfunction in 56 critical ill COVID-19 patients. No fatigue ICU, fatigue ICU, no fatigue follow-up, and fatigue follow-up, reflects fatigue status and whether the CNS biomarkers were collected at the ICU or at follow-up. (**a**) The axonal biomarker NfL was significantly higher during the acute illness and normalized after 3–4 months. High levels of NfL were not associated with cognitive dysfunction. (**b**) The neuronal biomarker t-tau levels were slightly higher on average at follow-up, but any differences were minor. Indicating that neuronal damage was not increased during critical COVID-19, and was not associated with cognitive dysfunction. (**c**) The glial biomarker GFAp was higher during the acute critical COVID-19 and in patients with at least mild cognitive dysfunction at follow-up. (**d**) The glial biomarker GFAp showed significant effects on both age and gender. *NfL* Neurofilament light chain. *T-tau* total tau. *GFAp* Glial fibrillary acidic protein. *ICU* intensive care unit. *MoCA* Montreal cognitive assessment score.

The concentration of the glial biomarker GFAp at follow-up was higher in patients with at least mild cognitive dysfunction (*p* = 0.01) compared to no cognitive dysfunction. GFAp concentration was also higher during acute COVID-19 compared to at follow-up. Concentration of GFAp increased with age and was higher in women than in men (Fig. 7).

NfL levels were not associated with cognitive dysfunction. There was however an association between increased concentration and age (*p* < 0.001).

The neuronal biomarker t-tau concentration was higher at follow-up (*p* = 0.01), but the differences were minor. Increased levels of t-tau were not associated with cognitive dysfunction.

## Discussion

The main finding of this study was that elevated GFAp during acute COVID-19 is associated to the development of mild cognitive dysfunction at follow-up, especially in women, following critical COVID-19.

GFAp is the main intermediate filament and the main protein in mature astrocytes, but has also been shown to be expressed in peripheral glia^23^, Schwann cells^24^ and non-CNS cells^25^. It maintains the mechanical strength and shape of the cell. However, the exact function of GFAp is not known. GFAp and the astrocytes are believed to have a protective function in hypoxia and to maintain normal CNS myelination. GFAp expression is induced by CNS damage and degeneration, leading to reactive gliosis^26^. The expression increases with older age^27,28^, especially in the hippocampus (which is the most sensitive area to reactive gliosis), as well as in the frontal and temporal cortex^29^. This is in line with our findings with increasing GFAp values with increasing age. Elevated GFAp has previously been correlated to cognitive decline and lower mini mental state examination score while assessing Alzheimer’s disease^30^. Regarding critically ill COVID-19 patients, it is still unknown whether there is direct damage to the CNS from the virus infection or whether it is multifactorial genesis due to hypoxia, coagulopathy etc. In a study of 47 patients with moderate (hospitalized requiring supplemental oxygen) and critical (admitted to the ICU) COVID-19, NfL and GFAp were elevated at onset of symptoms and after 11 days, with an early peak of GFAp and sustained elevated levels of NfL, showing evidence of neuronal injury and glial activation^18^. The initially elevated concentrations of GFAp and NfL normalised regardless of disease severity or persisting neurological symptoms at follow-up after 6 months, suggesting that the neurological sequelae are not necessarily accompanied by ongoing CNS injury^19^. GFAp has previously been suggested as a biomarker for predicting CIN and CIM^2^. We found that lower levels of GFAp in plasma were associated to the development of physical fatigue, mental fatigue and reduced motivation at follow-up. While men have higher mortality in acute COVID-19 disease, women have previously been shown to be more prone to fatigue after COVID-19 infection^31^. We found that the GFAp decrease was more pronounced in women, specifically in those with physical fatigue. This is a very interesting observation that will have to be verified by replication in future studies. Physical fatigue is not an isolated neuropsychiatric disorder in patients after treatment in the ICU for critical disease and the reasons for fatigue could be the multifactorial. COVID-19 is a respiratory disease and many have long-term reduction in lung function and other sequelae from their ICU stay. Nevertheless, in our setting, the fact that patients with physical fatigue had lower GFAp concentrations could suggest that at least some of the symptoms are indeed related to neurological effects. Regarding mental fatigue, we found that men showed a more pronounced GFAp decrease in those affected. This is contrary to previous studies and these findings have, to our knowledge, never previously been described. The mechanisms behind these findings are not obvious, and it is possible that they could be due to selection bias for example in ICU-admission policy. Additionally, GFAp concentrations in our study were generally low compared to other studies investigating CNS biomarkers in COVID-19^18,32^. Furthermore, fatigue is subjective and could be difficult to determine with high certainty. The use of MFI-20 has however been used to determine fatigue in a multitude of different diseases and populations, including the ICU setting^33^. Similarly, MoCA has been widely applied to determine cognitive impairment. The cut-off value of 26 points has been criticized for being too strict resulting in false positive results, and a cut-off value of 23 points has been suggested^34^. We chose a cut-off of 26 points in this study in order not to increase the risk of false negatives in regards to cognitive impairment after COVID-19 infection.

The NfL concentrations were increased in the acute phase and then normalized at follow-up, which is in line with previous findings^19^. This is indicative of acute axonal injury that is not persisting and not related to ongoing fatigue or cognitive impairment. However, our results show that lower values of NfL at ICU and at follow-up are associated with reduced motivation which is opposite to findings in previous studies. In other conditions there is evidence of the prognostic value of NfL in both neurological and in non-neurological diseases. Specifically, increased NfL has been found to be suggestive of poor outcome in cardiac arrest^35^, stroke^36,37^ and traumatic brain injury^38^. It has also been suggested as a biomarker for cognitive impairment and delirium after elective surgery^39^.

We found that T-tau was not associated with cognitive impairment nor fatigue at follow-up. However, the concentration was higher on average at follow-up, indicating that neuronal damage was not increased during acute critical COVID-19. A previous study has shown elevated t-tau in traumatic brain injury^40^. However, t-tau may be a less sensitive marker than serum NfL in CNS injury^41^.

The development of fatigue and cognitive impairment post-ICU have several potential causes. COVID-19 patients are diagnosed with ARDS and are at risk for PICS and ICU-acquired muscle weakness (ICUAW). It would be interesting to further investigate the relationship between CNS biomarkers and in particular GFAp in the context of PICS and ICUAW in non-COVID-19 patients. In our follow-up group 10.9% of the patients were diagnosed with CIN/CIM. There is evidence that cognitive impairment in areas such as executive function, memory, processing speed, persisting attention, and psychomotor tasks lasts for years after critical illness^42–44^ and that it could be due to hypoxia^44^. However, in our cohort the investigation of CIN/CIM with electroneuro/myography was not systematically performed and the number of patients could be higher. Therefore, it is possible that CIN/CIM could be a contributing factor to the development of fatigue in this population. A high number of patients experience cognitive dysfunction following ARDS^45^, with as many as 80–100% of patients being affected at hospital discharge declining to 20% at 5 years^46,47^. Additionally, being subject to mechanical ventilation is in itself associated with persistent cognitive impairment^48^. It would indeed be interesting to further study whether GFAp could predict neuropsychiatric outcomes in that broader group of ICU patients. Furthermore, it could help distinguish whether the increased biomarkers are due to the direct impact of the virus on the CNS or if it is due to complications of ARDS such as hypoxemia, muscle relaxant, critical illness etc.

### Strengths and limitations

The main strengths of the present study are the prospective design, and that biomarker analysis was performed both during acute COVID and at follow-up as well as being directly correlated to well-validated instruments for both fatigue and cognitive function.

The main limitation of this study is the lack of a control group of ICU patients without COVID-19 infection. However, very few patients admitted to the ICU without COVID-19 were available during the pandemic. Furthermore, the patients in this study have gone through a long-term ICU stay with a critical disease, and that in itself could affect the long-term psychiatric outcome making it difficult to categorically say that the symptoms are caused by COVID-19. The cutoff values for MFI-20 are based on a historic study in another population, we used the 95th percentile of MFI-20 as the cut-off, which means that patients with mild fatigue could be undetected. We have adjusted for age and sex as confounding factors and there were no significant difference in background characteristics or discharge parameters between the groups with and without fatigue or cognitive impairment respectively, however, residual confounding factors cannot be excluded in this relatively small cohort.

## Conclusion

**Figure 8:**
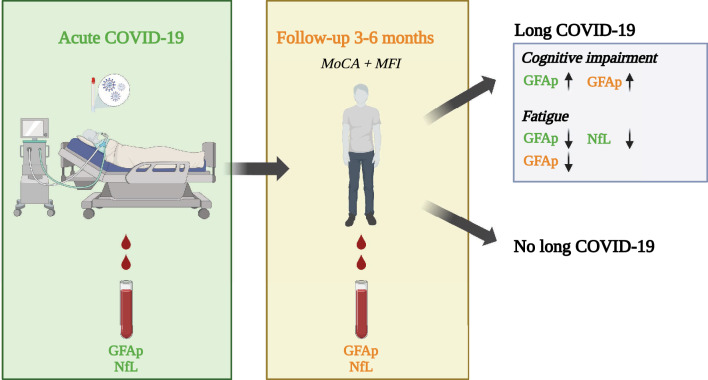
Graphical abstract of the study. Patients with acute COVID-19 admitted to the ICU were assessed with MoCA and MFI-20 at follow-up after 3–6 months. Both at the ICU and at follow-up analysis of CNS biomarkers GFAp and NfL were made and related to fatigue and cognitive impairment at follow-up. *ICU* intensive care unit. *GFAp* Glial fibrillary acidic protein. *NfL* Neurofilament light chain. *MoCA* Montreal cognitive assessment score. *MFI-20* Multidimensional Fatigue inventory.

Elevated GFAp concentrations during acute COVID-19 was associated with the development of mild cognitive dysfunction at follow-up after 3–6 months (Fig. 8), and this association was more pronounced in women. The findings suggest that circulating CNS biomarkers may be useful in predicting neuropsychiatric outcome after critical COVID-19 and indicate the need for targeted rehabilitation post-ICU.

## Data Availability

Data cannot be made publicly available because of Euroean and Swedish privacy and patient protection laws, but is available through the SciLifeLab data repository after securing ethical permission and appropriate data access agreements (https://doi.org/10.17044/scilifelab.14229410).
